# The Nonconventional MHC Class II Molecule DM Governs Diabetes Susceptibility in NOD Mice

**DOI:** 10.1371/journal.pone.0056738

**Published:** 2013-02-13

**Authors:** Marc A. J. Morgan, Pari S. S. Muller, Arne Mould, Stephen A. Newland, Jennifer Nichols, Elizabeth J. Robertson, Anne Cooke, Elizabeth K. Bikoff

**Affiliations:** 1 Sir William Dunn School of Pathology, University of Oxford, Oxford, United Kingdom; 2 Department of Pathology, University of Cambridge, Cambridge, United Kingdom; 3 Wellcome Trust - Medical Research Council Cambridge Stem Cell Institute, Council Cambridge Stem Cell Institute, University of Cambridge, Cambridge, United Kingdom; Oklahoma Medical Research Foundation, United States Of America

## Abstract

The spontaneous destruction of insulin producing pancreatic beta cells in non-obese diabetic (NOD) mice provides a valuable model of type 1 diabetes. As in humans, disease susceptibility is controlled by the classical MHC class II genes that guide CD4^+^ T cell responses to self and foreign antigens. It has long been suspected that the dedicated class II chaperone designated HLA-DM in humans or H-2M in mice also makes an important contribution, but due to tight linkage within the MHC, a possible role played by DM peptide editing has not been previously tested by conventional genetic approaches. Here we exploited newly established germ-line competent NOD ES cells to engineer a loss of function allele. DM deficient NOD mice display defective class II peptide occupancy and surface expression, and are completely protected against type 1 diabetes. Interestingly the mutation results in increased proportional representation of CD4^+^Foxp3^+^ regulatory T cells and the absence of pathogenic CD4^+^ T effectors. Overall, this striking phenotype establishes that DM-mediated peptide selection plays an essential role in the development of autoimmune diabetes in NOD mice.

## Introduction

In mice and humans, susceptibility to type 1 diabetes is predominantly controlled by the classical MHC class II loci responsible for positive and negative selection of CD4^+^ T cell clones during thymic development [1]–[3]. The diabetes-associated NOD I-A^g7^ molecule shares with predisposing human HLA-DQ alleles exceptional substitutions at the highly conserved Pro 56 and Asp 57 residues. This structural change creates an unusually wide peptide-binding groove [4]–[6]. The question how these unique features influence occupancy by self peptides and stimulate recruitment of autoreactive CD4^+^ T pathogenic effectors to pancreatic tissue has been extensively studied. An attractive idea is that the intrinsic instability of loosely bound peptides allows autoreactive CD4^+^ T cells to escape thymic deletion.

Besides highly polymorphic MHC class II subunits, surface display of diverse peptide ligands also depends on the combined activities of two dedicated chaperones, namely the conserved Invariant (Ii) chain and the nonconventional class II molecule DM required at distinct stages during maturation and export. Coassembly with the Ii chain prevents irreversible misfolding or aggregation, protects the nascent empty groove from association with ER quality control chaperones, and promotes trafficking to endosomal compartment(s). Here associations with the nonclassical class II molecule DM facilitate removal of Ii chain cleavage fragment(s). DM also stabilizes empty class II and acts sequentially on recyling class II to catalyze selection of best fit peptide ligands [7]. The NOD I-A^g7^ has an exceptional ability to spontaneously release the Ii chain derived CLIP peptide at acidic pH and may therefore be more accessible to peptide ligands within endocytic compartments [8]. On the other hand, human disease association studies suggest autoimmune disorders closely correlate with class II allelic products that lack intrinsic stability and are poor DM substrates [9]–[11]. Susceptibility to DM editing was recently shown to modulate insulin-specific T cell reactivity in mice [12].

Our recent studies demonstrate that peptide acquisition by the diabetogenic I-A^g7^ molecule in NOD mice depends on its Ii chain association. Interestingly Ii chain loss of function mutants display defective I-A^g7^ export and complete protection against type 1 diabetes [13]–[14]. At least 20 regulatory loci with the ability to influence disease have been previously characterized in NOD mice. Even though we backcrossed the Ii chain null allele onto the NOD background, set up homozygous matings at the 10^th^ backcross generation, and confirmed the presence of all previously described linkage markers associated with NOD derived recessive Idd loci necessary for the onset of disease by microsatellite analysis, we cannot rule out the possibility that a closely linked resistance locus may contribute to protection.

ES cell technology provides a powerful tool for studying autoimmune disease. Gene targeting is routinely achieved in the context of 129 and C57BL/6 genetic backgrounds, but efforts to derive germ-line competent ES cells from NOD and other refractory strains have only recently met with success due to the use of small molecule inhibitors [15]–[16]. Additionally homologous recombination in ES cells generally requires construction of an isogenic targeting vector. However the DMalpha locus in NOD mice is embedded within a long stretch of MHC sequences shared with the BALB/c (H-2^d^) strain [17]–[18]. To create DM-deficient NOD mice, here we exploited the BALB/c targeting vector and Southern screen previously used to demonstrate DM requirements in BALB/c mice [19]. This strategy allowed production of genetically pure DM-deficient NOD mice that are defective in class II peptide acquisition and CD4^+^ T cell maturation and completely protected against type I diabetes. The present results conclusively demonstrate that the dedicated class II chaperone DM governs diabetes susceptibility in NOD mice.

## Results

**Figure 1:**
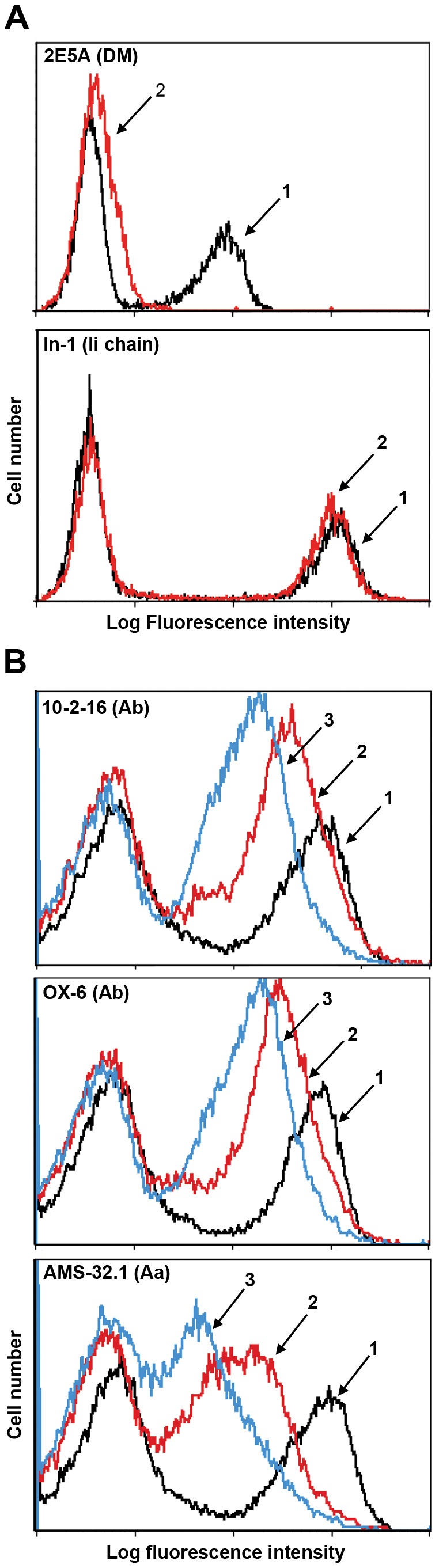
DM loss in NOD mice results in decreased I-A^g7^ surface expression. (A) Saponin-permeabilized splenocytes from wildtype (1) or DM mutant (2) NOD mice were incubated with anti-DM or anti-Ii chain mAb and FITC-conjugated anti-rat IgG (H+L) and analyzed by FACS. The DMα mutation eliminates DM and has no effect on Ii chain expression. (B) Splenocytes from (1) wildtype, (2) DM-deficient, or (3) Ii chain mutant NOD mice were stained with biotin-conjugated mAbs as indicated, followed by FITC-conjugated avidin. The shifts were detectable with conformationally dependent mAbs directed against distinct epitopes contributed by both chains and thus reflect decreased expression rather than a serological change. Representative data from one of three identical experiments with similar outcomes are shown.

**Figure 2:**
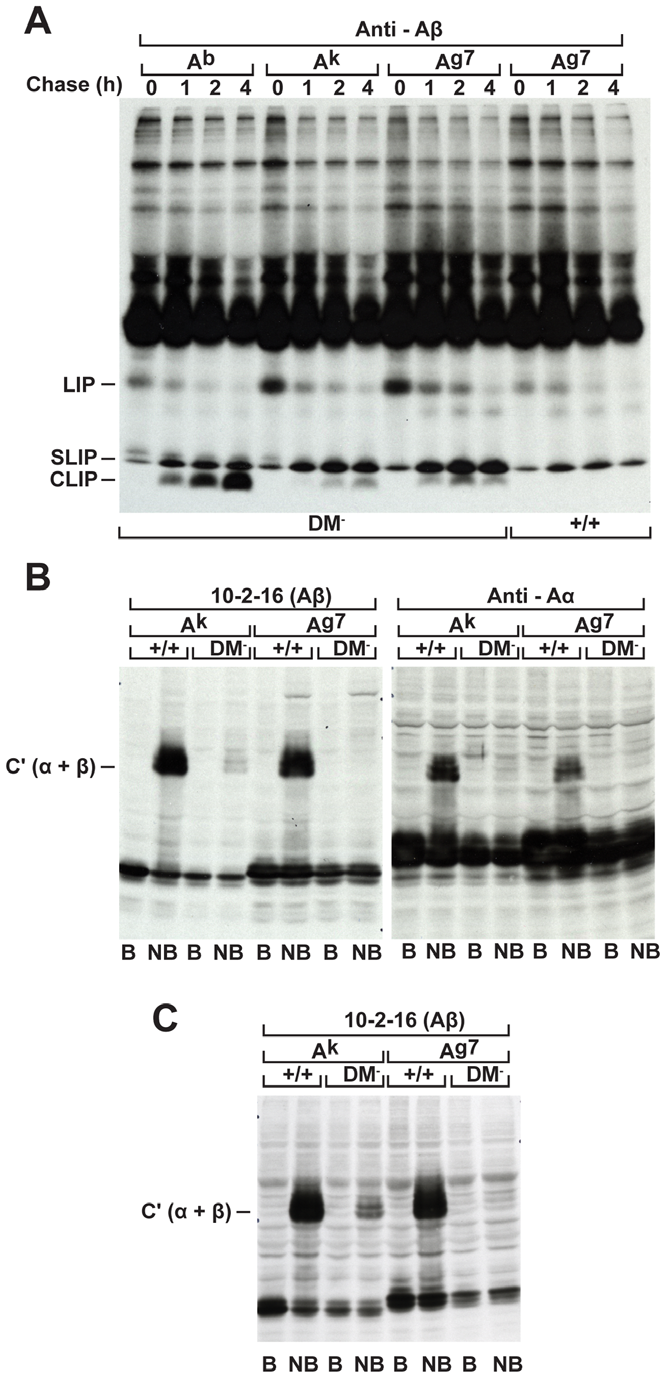
DM mutant NOD mice weakly express A^g7^/CLIP complexes and entirely lack mature compact A^g7^ dimers. (A) To evaluate occupancy by the Ii chain-derived CLIP peptide, DM mutant spleen cells expressing either the b or k haplotype, DM loss of function and wildtype NOD splenocytes were compared. Lysates were prepared from spleen cells pulsed for 40 mins with ^35^S-methionine and chased for different times as indicated and extensively pre-cleared with Rabbit anti-mouse IgG (H+L) Abs before immunoprecipitation with beta chain-specific Rabbit Ab. Complexes were boiled for 10 mins and analyzed on 10% polyacrylamide gels. (B) Spleen cell lysates were boiled (B) or kept on ice (NB) and samples resolved on 10% gels under reducing conditions, transferred to nitrocellulose membranes, and Western blots probed with 10-2-16 or alpha chain-specific Rabbit Ab as indicated. (C) Western blot analysis of lysates prepared from LPS-stimulated BMDCs.

DM loss in b haplotype mice causes accumulation of A^b^/CLIP complexes stably expressed on the cell surface [20]–[22]. In contrast here, targeted disruption of the DM alpha locus in NOD mice results in markedly decreased I-A^g7^ surface expression (Figure 1). Next we examined class II maturation defects in pulse-chase experiments. As expected in boiled samples, I-A^g7^ expressed in the absence of DM readily dissociates and CLIP is detectable ahead of the dye front (Figure 2A). However in contrast to long-lived A^b^/CLIP complexes present after 4 h of chase, we observed barely detectable levels of A^g7^-associated CLIP. As for A^k^/CLIP, A^g7^/CLIP complexes have a short half life.

To directly evaluate class II peptide occupancy at steady state, we examined constitutive expression of mature compact A^g7^ dimers via Western blot analysis. Analysis of non-boiled samples confirms the presence of a substantial pool of mature compact dimers produced by wildtype splenocytes (Figure 2B) and LPS-treated bone marrow derived dendritic cell cultures (Figure 2C). In striking contrast under the same conditions, A^g7^ produced by DM mutants entirely lacks SDS stability. Similar conclusions were reached using mAbs reactive with distinct epitopes or conformationally independent chain specific rabbit Abs. Thus, the mutation disrupts selection of tightly bound self peptides.

**Figure 3:**
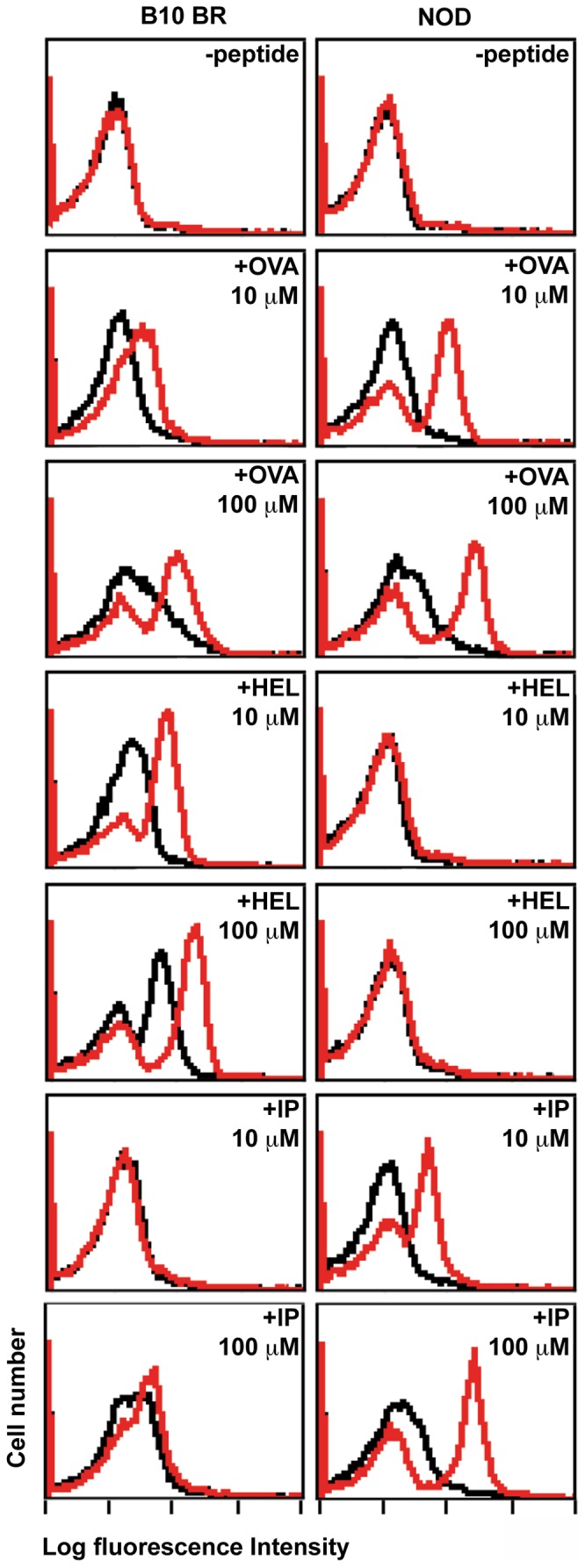
DM mutants display functionally empty I- A^g7^ molecules with markedly enhanced peptide-binding capabilities. DM mutant (shown in red) and control wildtype (shown in black) splenocytes from B10.BR or NOD mouse strains were cultured for 5 hrs at 37°C with biotin-conjugated peptides or medium alone as indicated, stained with FITC-labelled avidin, and analysed by FACS. OVA 323–339 preferentially binds to I- A^g7^, HEL 46–61 selectively binds to A^k^ molecules, whereas NOD DM mutants gain reactivity towards the lambda repressor cI peptide P12–26 (IP).

Additionally splenocytes were incubated with a panel of biotinylated peptides, stained with FITC avidin and analysed by flow cytometry. As expected H-2^k^ DM mutants display markedly enhanced peptide-binding capabilities, and unlike wildtype even bind OVA 323–329 (Figure 3). Similarly, NOD DM mutants gain reactivity towards lambda repressor cI peptide P12–26. In contrast, HEL46–61 exclusively binds to H-2^k^ and not DM-deficient NOD splenocytes. DM loss creates a pool of functionally empty I-A^g7^ molecules readily available to exogenous ligands (Figure 3).

Presentation of self peptides by subsets of thymic stromal cells governs T cell repertoire selection [23]–[24]. Class II expression by cortical epithelial cells mediates positive selection of mature CD4^+^T cells, whereas self antigens in the medulla induce central CD4^+^ T cell tolerance [25]–[26]. DM loss in b haplotype mice profoundly disturbs CD4^+^ T cell development [20]–[22] whereas relatively efficient selection of mature CD4^+^ T cells has been described for H-2^d^ and H-2^k^ mutants [19], [27]. These strain differences potentially reflect isotype-specific chaperone requirements since I-E functional activities remain intact. In contrast, NOD mice exclusively express I-A^g7^ [28].

**Figure 4:**
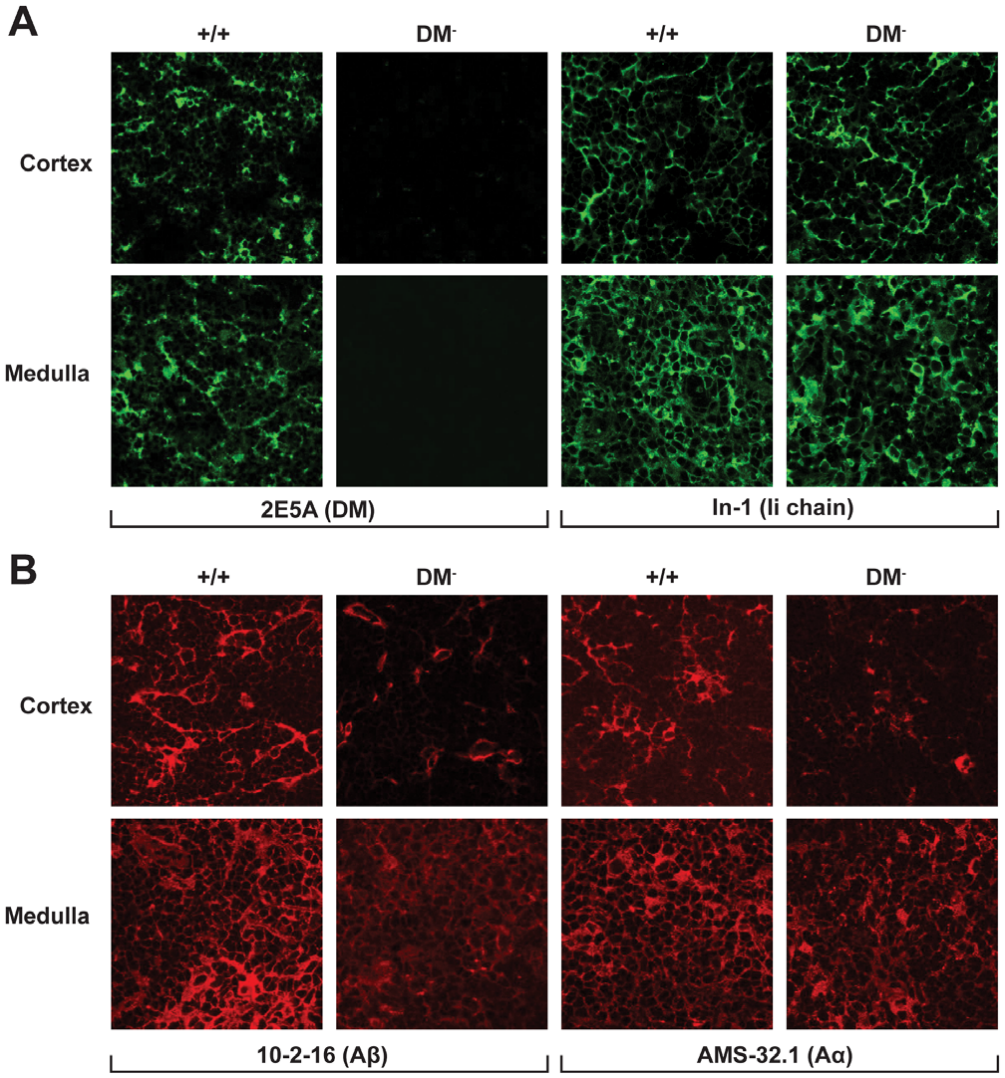
DM functional loss results in decreased A^g7^ expression within the thymic cortical epithelium and medullary regions. Acetone-fixed cryostat sections of thymic tissue were stained with the indicated mAbs.

To investigate DM contributions to CD4^+^ T cell development, we initially examined class II expression in thymic cryosections. As shown in Figure 4, our targeted deletion completely eliminates DM but has no obvious effect on Ii chain expression throughout the thymus. The mutation leads to decreased class II expression within the cortex and medullary regions. Importantly the 10-2-16 mAb recognizes not only mature I- A^g7^ compact dimers but also has substantially reactivity with free β chains (see Figure 2). Thus a strong argument can be made that these results do not simply reflect DM-dependent conformational changes. It will be important to learn more about DM contributions guiding presentation of self peptides by the various subsets of thymic stromal cells responsible for positive and negative selection of mature CD4^+^ T cells.

**Figure 5:**
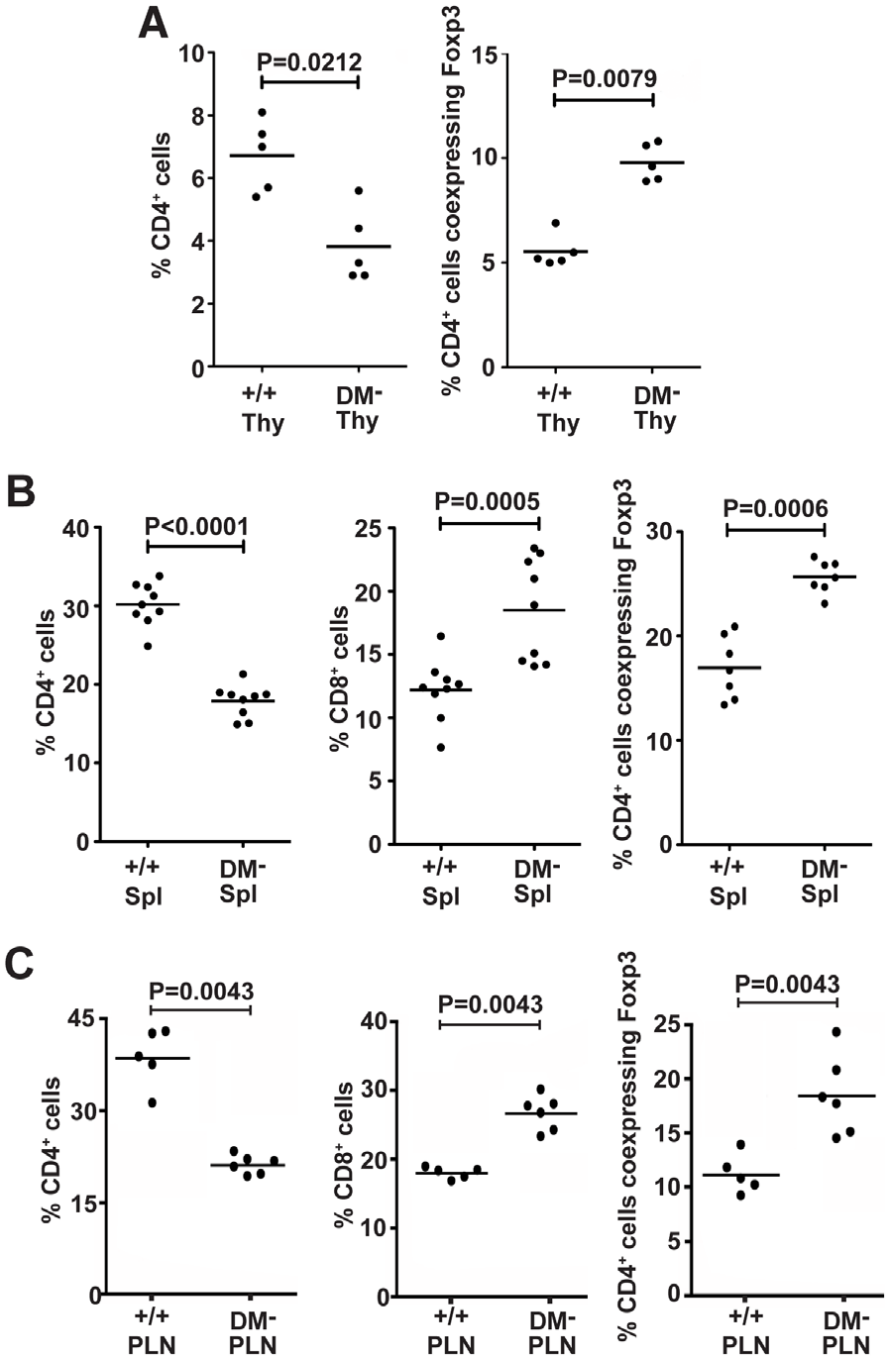
DM functional loss favours development of the CD4+ Foxp3+ T cell subset. Thymus (A), spleen (B), and pancreatic lymph node (C) cell suspensions were stained for CD4, CD8, and Foxp3 expression and analysed by flow cytometry.

To evaluate CD4^+^ T cell development, we analysed T cell subpopulations using flow cytometry. As for Ii chain loss in NOD mice [14], we also consistently observe here significantly increased proportional representation of CD4^+^ Foxp3^+^ T cells in DM deficient NOD mice in the thymus and periphery (Figure 5A–C). There was also a significant shift in the representation of mature CD4^+^ and CD8^+^ T cell subsets. DM mutants display decreased CD4^+^ T cells and comparable increases in percentages of mature CD8^+^ T cells (Figure 5A–C). Importantly, this developmental bias is unaccompanied by changes in class I expression (data not shown).

**Figure 6:**
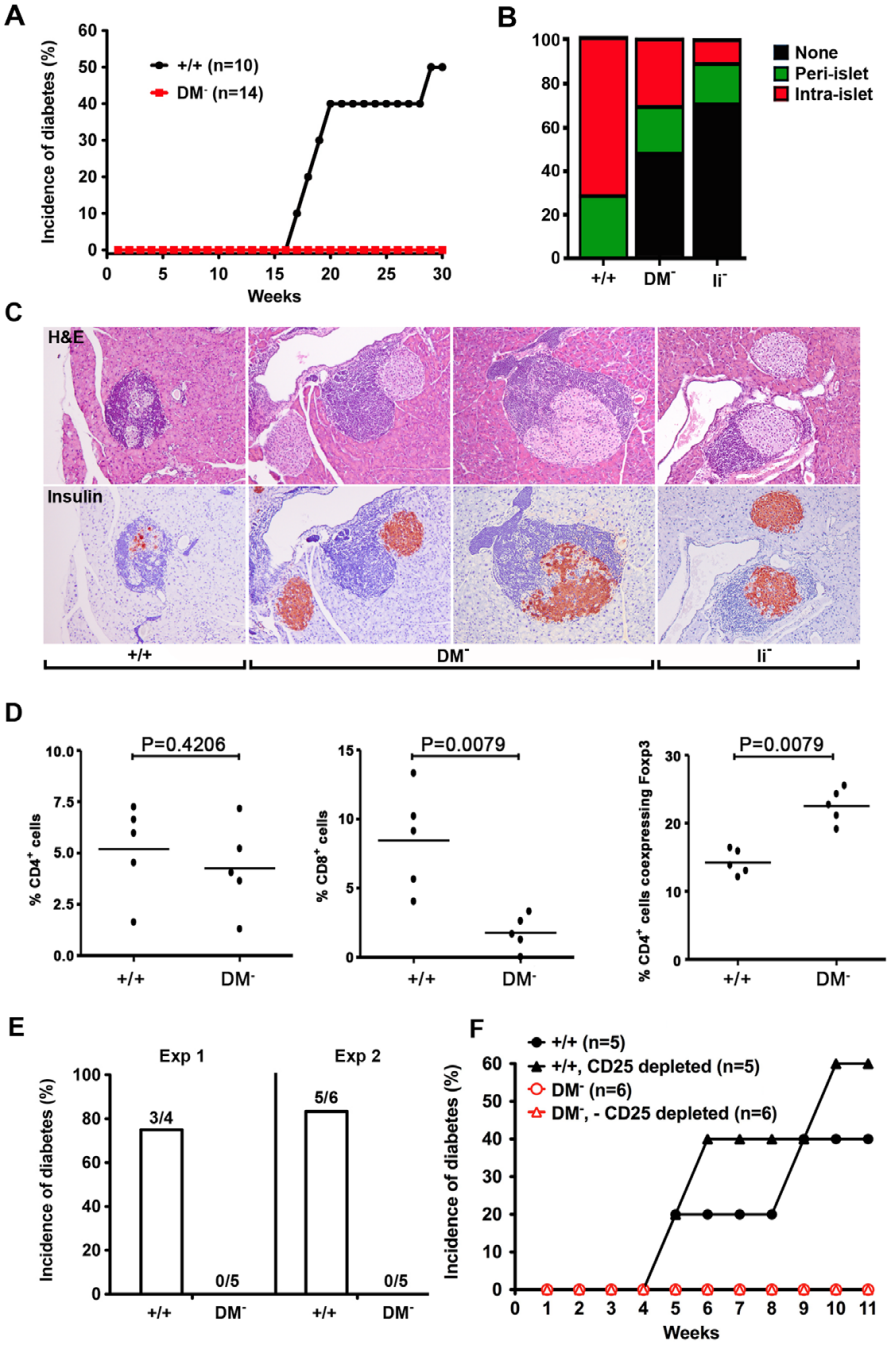
DM mutant NOD mice protected against autoimmune diabetes fail to develop pathogenic CD4^+^ T effectors. (A) The onset of diabetes was evaluated by measurement of blood glucose levels in age-matched DM mutant and wildtype females. The incidence of diabetes has been absolutely zero over the past 2 years with parents in homozygous mutant breeding cages routinely tested at 6 mo of age. (B) The percentage of islets with a given degree of infiltration at 5 mo of age. (C) Typical examples of islet architecture assessed by H & E and insulin staining demonstrate substantial destruction by intra-islet infiltrates in wildtype mice, the normal islets predominantly seen in Ii chain mutants, and benign peri-islet infiltrates present in DM deficient NOD mice. (D) The representation of CD4, CD8, and Foxp3^+^ T cell subsets in pancreatic infiltrates was analyzed by flow cytometry. (E) DM-deficient NOD mice are resistant to cyclophosphamide-induced type 1 diabetes. Blood glucose levels were measured in age-matched wildtype and mutant females injected i.p. with cyclophosphamide at a dose of 200 mg/kg. (F) Depletion of CD25^+^ T cells fails to reveal the presence of pathogenic CD4^+^ T effectors in DM mutants. NOD.scid females were reconstituted with the indicated T cell populations.

Islet autoantigen-specific CD4^+^ T cells play a pivotal role in type 1 diabetes [29]–[30]. To test whether DM functional loss disrupts recruitment of inflammatory effectors to pancreatic islets and/or progression to overt disease, we initially measured blood glucose levels. As expected the onset of disease in wildtype females begins at approximately 15 weeks and within our facility approximately 50% were diabetic by 30 weeks. In striking contrast over the same time interval, DM mutants remain completely asymptomatic (Figure 6A). Histological examination demonstrates substantially less intra-islet infiltration (Figure 6B) and as judged by insulin staining, little evidence for beta cell destruction (Figure 6C). Interestingly despite increased percentages of peripheral CD8^+^ T cells in the spleen (Figure 5B) and pancreatic lymph nodes (Figure 5C) there was markedly less recruitment to pancreatic islets (Figure 6D). Additionally cyclophosphamide, an alkylating agent known to shift the Teffector/Treg balance and accelerate diabetes onset in wildtype NOD [31] fails to induce diabetes in DM mutants (Figure 6E). Consistent with the idea that the protective mechanism does not simply reflect more effective Treg activities, we also observe that unlike wildtype, DM mutant splenic T cells lack the ability to transfer diabetes to NOD.scid recipients (Figure 6F). Moreover, the inability to transfer disease was not reversed by CD25 depletion (Figure 6F). Collectively these results strongly argue that DM function is required for the development of CD4^+^ pathogenic effectors and demonstrate for the first time a causal relationship between DM-mediated peptide selection and autoimmunity.

## Discussion

The NOD I-A^g7^ and human HLA-DQ8 molecules, strongly associated with diabetes, both lack a conserved aspartic acid at position β 57 that normally pairs with the conserved arginine α 76 [4]–[6]. The loss of this salt bridge widens the P-9 pocket and creates a positively charged surface that seems to select peptides with acidic residues at the P-9 position [32]. However in contrast to these conclusions reached in peptide elution experiments, studies aimed at identification of autoantigens recognized by pathogenic T cells in type 1 diabetes suggest that naturally processed islet peptides may bind to I-A^g7^ in an atypical manner [33]. Considerable data argue that CD4^+^ T cells in humans and NOD mice recognize common pancreatic beta cell antigens [34]. DM has been shown to influence register shifting of insulin peptide binding to I- A^g7^ [12] and HLA-DR4-restricted presentation of the immunodominant epitope of GAD [35].

Recent studies suggest that DM selectively binds to partially empty class II [36] but of course in vivo under normal physiological conditions the vast majority of newly synthesized class II coassembled in the presence of excess Ii chain initially retains CLIP occupancy. In contrast to competitive class II interactions with Ii chain, DM, and peptide ligands inside professional antigen presenting cells, a substantial body of work demonstrates that the empty class II groove has a remarkable degree of flexibility. For example in early studies a synthetic peptide containing only two residues from the original HA epitope stably bound to the HLA-DR1 molecule [37]. Recent work even documented bidirectional CLIP peptide binding to HLA-DR1 [38]. The idea that nonconventional class II interactions with Ii chain and DM play a causative role in autoimmune disease is strongly supported by recent evidence that an alternative CLIP peptide associated with HLA-DQ2 is a poor DM substrate [9].

DM functional loss in b haplotype mice profoundly disturbs CD4^+^ T cell development [20]–[22]. Thus mature CD4^+^ T cells exclusively selected on A^b^/CLIP complexes gain reactivity towards natural self peptides presented by wildtype molecules. CD4^+^ T cell maturation is more severely compromised in double mutants lacking both Ii chain and DM [39]–[41]. In striking contrast, DM loss in k haplotype mice creates a substantial pool of empty or loosely occupied A^k^ molecules [27]. Similarly here DM mutant NOD mice display short-lived A^g7^/CLIP complexes, decreased A^g7^ surface expression and enhanced A^g7^ peptide binding activities. DM loss results in reduced percentages of mature CD4^+^ T cells and interestingly as for Ii chain-deficient mice expressing diverse MHC haplotypes [14] an increased proportional representation of CD4^+^ Foxp3^+^ Tregs. However unlike the dominant protective mechanism described previously for Ii chain mutants, here depletion of Tregs failed to reveal the presence of pathogenic CD4^+^ effectors. Future studies will investigate whether DM-dependent peptide selection is required in the thymus for maturation of pathogenic CD4^+^ T cells and/or presentation of islet antigens for activation of autoreactive CD4^+^ T cells during the development of type 1 diabetes.

The present experiments demonstrate NOD ES cells can be successfully subjected to gene targeting culture conditions –i.e., drug selection and growth as single cell colonies and yet retain germline competence. Besides conventional class II and its dedicated chaperone DM, tightly linked genetic susceptibility determinants closely mapped within the MHC have also been implicated in autoimmune diabetes. Likely candidates include LMP and TAP genes encoding components of the class I peptide loading machinery. DO, another closely linked non-classical class II molecule, associates with DM to influence peptide selection in DC subsets and germinal center B cells and probably also dampens DM activities within thymic medullary epithelial cells [42]–[43]. Recent experiments suggest that DO controls peptide acquisition and diabetes susceptibility in NOD mice [44]. Ectopic expression resulted in reduced peptide occupancy and protection mediated by a Treg independent mechanism. These observations are reminiscent of early reports describing both central and peripheral tolerance mechanisms underlying diabetes prevention in gain of function MHC class II transgenic strains [45]. In contrast here, the DM null allele was engineered in the context of the unique MHC haplotype in NOD mice. It is now possible to similarly exploit NOD ES cells to examine possible influences of additional components of the MHC class I and class II peptide loading pathways under physiological conditions in genetically pure mutant mice using gene targeting approaches.

## Materials and Methods

### Animals

All animal procedures were approved by the Ethical Review Committees of the University of Oxford and University of Cambridge, and carried out under license from the Home Office. Animals were sacrificed by cervical dislocation (a Home Office approved Schedule One procedure).

Wildtype NOD/LtJ, B10.BR, and B.C-9, a strain congenic with C57BL/6 but expressing the Igh a allotype of BALB/c, were bred and maintained by brother-sister matings. The H-2^b^ DM alpha-deficient strain [20], as well as DM mutants carrying the H-2^k^ haplotype [27], and Ii chain deficient NOD [14] mice, have been previously described.

The targeting vector and Southern screen used for generation of DM alpha mutant BALB/c mice were previously described [19]. We exploited this completely homologous BALB/c vector and our proven Southern screening strategy to disrupt the DM alpha locus in the NOD mouse strain. Germ-line competent NOD ES cells were maintained in N2B27 medium (Stem Cell Sciences) supplemented with the mitogen-activated protein kinase kinase inhibitor PD0325901 and the glycogen synthetase kinase –3-inhibitor CHIR99021 (2i conditions) plus recombinant LIF (ESGRO; Millipore). Drug-resistant colonies selected in the presence of G418 and FIAU were picked and expanded into 96-well tissue culture trays, and split once; one set of duplicate trays was frozen and DNA prepared from the other tray was subjected to Southern blot analysis using restriction enzyme and probe combinations as described previously [19]. Trypsinization can adversely affect NOD ESC morphology leading to a non-adherent phenotype. Therefore we adopted a mild dissociation protocol. Cells were dissociated with PBS containing 1x trypsin, 1 mM EDTA, and 1% chicken serum (Sigma C5405). All trypsinization steps were performed at room temperature and monitored by microscopy (typically 2–5 minutes). Trypsin was quenched with DMEM containing 2% BSA (Sigma A3311) and cells were spun down to remove residual trypsin prior to resuspension in 2i medium. To generate chimeras, correctly targeted ES cell clones were injected into C57BL/6 blastocysts. Germ-line chimeric males were crossed to NOD/LtJ females to produce genetically pure heterozygous progeny that were subsequently intercrossed to obtain homozygous mutants. The PCR screen to distinguish wildtype and DM alpha mutant alleles has been described [19]. Two independent targeted clones were used to generate two independent homozygous mutant sublines and direct comparisons made in numerous experiments demonstrate DM-deficient mice derived from both clones gave indistinguishable results.

Blood glucose levels were measured using One touch test strips (Lifescan; Johnson & Johnson) and values greater than 250 mg/dl were considered positive for diabetes.

### Antibodies and peptides

FITC, PE, or biotin-conjugated CD3 (145-2C11), CD4 (RM4-5), CD8 (53-6.7), and purified Rat anti-mouse H2-DM (2E5A) were from BD Pharmingen. Purified Rat anti-CD74 Ii chain (In-1) was from Santa Cruz. FITC Goat F(ab’)2 anti-Rat IgG (H+L) was from Caltag. Anti-Foxp3 (clone FJK-16 s) was from eBioscience. Biotin-conjugated MHC class II mAbs AMS-32.1 and 10-2-16 were from BD-Pharmingen, and OX6 was from Serotec. For H2-DM and Ii chain cytoplasmic staining, spleen cell suspensions were treated with 10% formalin for 10 min at room temperature and extensively washed with PBS containing 0.1% saponin (Sigma), and all subsequent staining and washing steps were conducted at room temperature in the presence of saponin (0.1%) as described [13]. Intracellular Foxp3 staining was performed according to the manufacturer’s instructions (eBioscience). Stained cells were analyzed on a Becton Dickinson FACSCalibur and the data processed using FlowJo software (Tree Star).

The biotin-conjugated peptides OVA 323–339 (ISQAVHAAHAEINEAAGR), Hen egg lysozyme 46–61 (HEL46–61; NTDGSTDYGILQINSR), and bacteriophage lamda repressor cI peptide P12–26 (LEDARRLKAIYEKKK) were purchased from Quality Controlled Biochemicals (Hopkinton, MA). FITC-labelled avidin D was from Vector Laboratories.

### Radiolabeling, immunoprecipitation, and Western blot analysis

Biosynthetic labelling, immunoprecipitation, and SDS-PAGE were conducted as previously described [27]. Briefly spleen cells were washed with warm HBSS containing 2% FCS and antibiotics and resuspended (2×10^7^/ml) in warm methionine-free DMEM supplemented with 4 mM glutamine and 5% dialyzed FCS. After 1 h at 37°C [^35^S]methionine was added (250 µCi/ml) for 40 min. The cells were either immediately harvested or subsequently re-suspended in a 5-fold excess volume of warm DMEM containing 15% FCS and a 10-fold excess of cold methionine, and incubated 1, 2, or 4 hours at 37°C and then harvested and washed twice with ice cold PBS. The cell pellet was lysed in buffer containing 1% Nonidet P-40, 20 mM Tris-HCl (pH 7.5), 150 mM NaCl, 5 mM EDTA, 1 mM PMSF, and 10 µg/ml aprotinin. After incubation on ice for 15 min, extracts were cleared of nuclei and debris by centrifugation for 30 min at 15,000 rpm. Lysates were precleared once with rabbit anti-mouse IgG (H + L) Abs (Zymed), twice with rabbit anti-rat IgG(H + L) Abs (Zymed), and twice with protein A-agarose (Life Technologies) before the addition of specific Abs. Immunoprecipitates were washed three times with buffer containing 0.05 M Tris-HCl (pH 8), 0.45 M NaCl, 0.5% Nonidet P-40, 0.05% sodium azide, and 1 µg/ml aprotinin, and then solubilized in Laemmli buffer containing 2% SDS and 2-ME by heating at 100°C for 10 mins. Samples were analyzed by SDS-PAGE, subsequently treated with Amplify (GE Healthcare), dried and exposed to x-ray film.

For Western blot analysis, sample buffer was added to post-nuclear detergent extracts and lysates were boiled for 5 min (B) or kept on ice (NB) before fractionation on 10% polyacrylamide gels. Proteins were transferred onto nitrocellulose membranes (Schleicher & Schuell) for 2 hr at 500 mA. Blots were rinsed in TBS-T then incubated overnight in TBS-T with 10% dry milk and 3% BSA followed by one rinse before the addition of primary Abs diluted in TBS-T containing 5% BSA and 5% calf serum. After a 60 min incubation, blots were extensively washed with TBS-T containing 1% BSA followed by a 30 min incubation in secondary Ab diluted in TBS-T containing 3% BSA. Preadsorbed HRP secondary Abs were donkey anti-rabbit Ig (cat number NA934V; Amersham) or sheep anti-mouse Ig (cat number NA931V; Amersham). Blots were washed with TBS-T and developed by chemiluminescence using ECL (cat RPN2106; Amersham).

### Confocal microscopy

Thymi were embedded in OCT and frozen in liquid nitrogen. Sections were mounted on glass slides and fixed by brief immersion in cold acetone. Sections were incubated for 1 hour at room temperature in PBS containing either 20% fetal calf serum (biotin-conjugated anti MHC class II abs) or 5% goat serum+0.5% BSA (rat mAbs) and washed prior to addition of primary abs listed above. Secondary Alexa Fluor 488 conjugated Goat anti-rat IgG (H+L) and Alexafluor 594-conjugated Streptavidin were from Invitrogen Molecular Probes. Sections were mounted with Vectashield (Vector Laboratories) and imaged on a Zeiss LSM 710 confocal microscope.

### Histology

Pancreatic tissues were fixed overnight at 4°C in 10% neutral buffered formalin, dehydrated through a graded ethanol series, embedded in paraffin wax and representative 5µ sections were stained with H&E and scored for the degree of cellular infiltration. Between 200 and 500 islets were scored as follows: Non-infiltrated; Peri-islet where infiltration is restricted; and Intra-islet where 20–100% of the islet is infiltrated. For insulin staining, de-waxed sections were blocked in PBS containing 10% goat serum prior to addition of guinea pig anti-porcine insulin antibody (Dako). Following overnight incubation and blocking of endogenous peroxidase activity, secondary peroxidase conjugated Donkey anti-guinea pig IgG (H + L) was added, and subsequently developed using NovaRED peroxidase substrate.

### Cyclophosphamide

Blood glucose levels were measured in age-matched wildtype and mutant females initially injected with cyclophosphamide at 10–12 weeks of age. Cyclophosphamide from Sigma (cat number C7397) was freshly prepared in PBS at 20 mg/ml immediately before i.p. administration.

### Adoptive transfers into NOD.scid recipients

NOD.scid mice were bred and maintained under barrier conditions in the Biological Services facility of the Department of Pathology, University of Cambridge. They received standard laboratory food and water ad libitum in microisolator cages with filtered air and were handled under sterile conditions in a laminar flow hood. Spleen cell suspensions were isolated from non-diabetic wildtype or DM- NOD females, and where indicated CD25^+^ T cells were depleted by staining with anti-CD25 PE (eBiosciences), followed by anti-PE microbeads (Miltenyi Biotec) and passage through an autoMACS Pro Separator. 2×10^7^ cells were injected i.v. into the lateral tail vein of 6 wk-old NOD.scid females. Urinary glucose levels were tested using Diastix every 7 days (Bayer). Recipients scored as diabetic had urinary glucose concentrations of greater than 55 mM on two occasions, separated by at least 7 days.

### Isolation of pancreatic infiltrating cells

Pancreatic tissues were collected in harvest buffer comprised of PBS supplemented with 5% fetal calf serum, 100 mM glucose, and a protease inhibitor cocktail (Roche). After digestion with Liberase CL (0.33 mg/ml, Roche) in PBS containing 10 µg/ml DNAse I (Sigma) and 10% fetal calf serum, lymphocytes were isolated using a 33% Percol gradient (GE healthcare).

### Statistics

Data were analyzed using two-tailed non-parametric Mann-Whitney U test (GraphPad Prism 5.0 software). Values of p are indicated in the Figures. Results were considered to be significant if p was less than 0.05.
